# CAR Chase: Where Do Engineered Cells Go in Humans?

**DOI:** 10.3389/fonc.2020.577773

**Published:** 2020-09-11

**Authors:** Simone Krebs, Megan M. Dacek, Lukas M. Carter, David A. Scheinberg, Steven M. Larson

**Affiliations:** ^1^Molecular Imaging and Therapy Service, Department of Radiology, Memorial Sloan Kettering Cancer Center, New York, NY, United States; ^2^Molecular Pharmacology Program, Sloan Kettering Institute, New York, NY, United States; ^3^Pharmacology Department, Weill Cornell Medicine, New York, NY, United States; ^4^Department of Medical Physics, Memorial Sloan Kettering Cancer Center, New York, NY, United States

**Keywords:** CAR T cells, TCR T cells, PET/CT, reporter gene, T cell trafficking

## Abstract

Chimeric antigen receptor (CAR) – and T-cell receptor (TCR) – modified T-cells are rapidly emerging as a viable treatment option for cancer patients. While initial clinical trials for these CAR T cells showed response rates of over 90% in some cases, retrospective studies have revealed a wide variability in patient responses as well as a significant proportion of patients relapsing after an initial response. In addition, patients often have severe adverse reactions to this therapy (e.g., cytokine release and neurologic syndromes). As a result, much research is still needed to be able to predict both therapeutic outcomes and possible toxicities. Furthermore, little success has been seen in treating solid tumors with engineered T cells and uncovering modes of failure is a topic of much research. Finally, little is known about the T cells’ pharmacokinetics after infusion into the patient, as standard methods of tracking the cells analyze peripheral blood and tumor biopsies – both of which lack spatiotemporal information. Herein, we propose that reporter gene-based imaging of engineered T cells in humans would be tremendously valuable in elucidating the fate of the transplanted T cells and would greatly facilitate clinical translation of new CAR and TCR technologies. Currently, there are no FDA-approved reporter genes and few methods have advanced to human studies. Herein, we outline current reporter gene approaches to track engineered cells *in vivo*, analyze why current reporter genes have not progressed into the clinic, and propose “rules” for designing a widely applicable reporter gene for use in humans.

## Introduction

Adoptive transfer of T cells for infectious and neoplastic diseases is the subject of intense clinical research. Fundamental approaches to live T cell immune therapy are the use of cells transduced with T cell receptors (TCRs) in which recognition of the tumor antigen occurs through presentation on cell surface human leukocyte antigens (HLA) and the use of chimeric antigen receptors (CARs) that typically are specified by a single-chain variable region domain of an antibody and directed to a cell surface tumor-associated antigen (1, 2). Over 500 clinical trials of CAR T cells are currently registered with the NIH.

**FIGURE 1:**
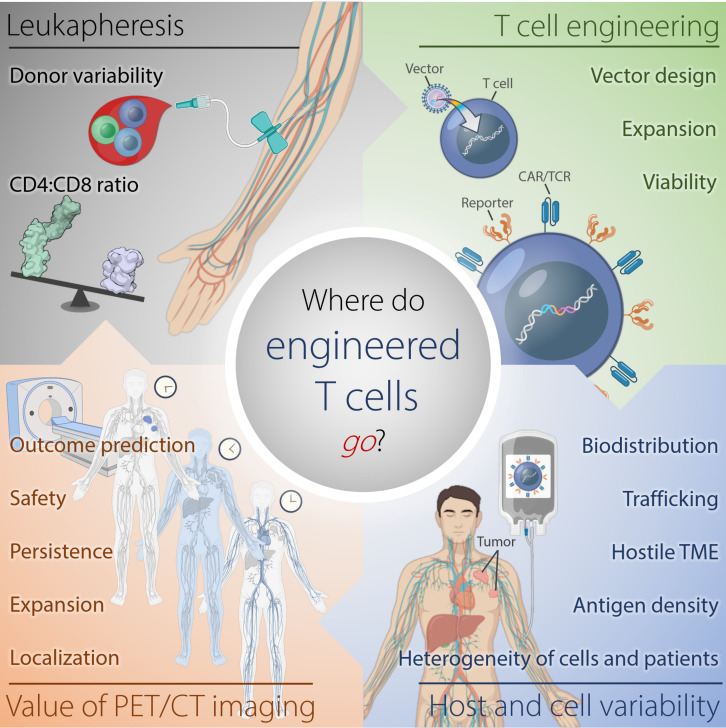
Where do engineered cells go in humans? This scheme depicts the complexity of factors affecting outcome of adoptive T cell therapy against tumors arising from harvesting of T cells, generation of the engineered T cells, and intra- and interindividual differences in the tumor-bearing host. Serial reporter-based molecular imaging can capture changes in the whole body non-invasively and in real-time, thus, potentially improving safety and enabling outcome prediction. TME, tumor microenvironment.

These cell therapies have demonstrated highly variable efficacies and sometimes severe and fatal toxicities. Therefore, understanding why certain cell therapies succeed, while others fail to deliver major response rates or durable clinical responses in patients, would be improved by monitoring of the infused immune cell trafficking, homing to the tumor site, retention, expansion, and engagement with the target cells (Figure 1). Traditional pharmacokinetic studies such as those used with drugs, are not feasible and do not describe the distribution of these living, proliferating agents, which may persist for years. Cell therapies are highly complex, individually manufactured for each patient, and are affected by prior therapy and disease burden, among other factors. Therefore, the availability of a biomarker or tool for continuous non-invasive monitoring in real-time and in the whole body would be invaluable. However, so far, no such biomarker or tool exists.

Current methods to monitor effects of cells infused into patients involve serum profiling of cytokines associated with T cell activation, direct enumeration of tumor-specific T cell numbers in blood, and tumor biopsies. These approaches do not provide the needed real time dynamic and spatial information about the transferred T cells in the body. Moreover, repeated biopsies are neither feasible nor ethical. Hence, clinicians remain blind as to whether the changes in T cell numbers in the blood relates to expansion at the primary tumor site, metastatic foci, or at off-tumor sites. Direct visualization of T cell trafficking *in vivo* could map their biodistribution, accumulation at the tumor site or other tissues, expansion, contraction and persistence in real time and in the whole body. Such an imaging surrogate could evolve into prognostic biomarkers that could provide important data about potential failure or success of a therapy, or re-dosing, thus enabling clinical decision making at an early time point during the therapy course. Ultimately, such a tool could facilitate and accelerate pre-clinical and early clinical development of novel T-cell based cancer therapies, vastly reducing the time and cost of development, and sparing patients unneeded toxicity.

## Principles of Cell Tracking

Direct *ex vivo* labeling of cells, introduced for tracking of human white blood cells in the 1960’s and 70’s, is relatively simple and does not involve genetic modification of the cell (3–5). Blood was harvested, labeled with [^111^In]In-oxine, and re-infused to detect infectious and inflammatory sites. Later, tumor-infiltrating lymphocytes (TILs) were harvested from human tumors, labeled with [^111^In]In-oxine and scintigraphy showed migration of the labeled TILs and accumulation at sites of melanoma metastases (6). These initial studies demonstrated the proof-of-principle for tumor-targeting of adoptively transferred T cells in patients and were confirmed in follow-up studies. However, a major disadvantage of this technique is that the label is diluted over time when cells divide, resulting in a rapid loss of label per individual cell. Furthermore, the label can be distributed asymmetrically to the progeny during cell division. These factors severely limit the imaging period and do not enable imaging of expanding cell populations.

**FIGURE 2:**
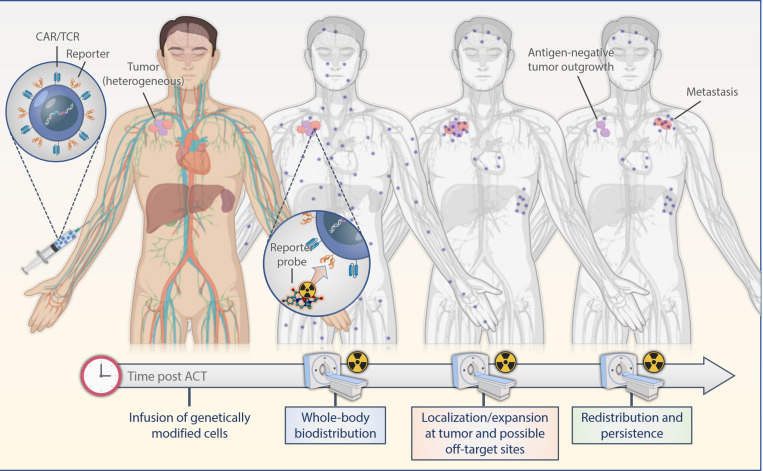
Scheme of reporter-based molecular imaging. If the expression of the reporter gene is stable, labeled cells can be observed over their entire lifetime allowing for extended longitudinal studies upon serial infusions of reporter-specific probes with PET or SPECT in the whole body. ACT, adoptive cell transfer.

Indirect labeling methods use a reporter gene that is introduced into the cell genome and translated into stable expression of an enzyme, receptor or a protein in conjunction with corresponding radiolabeled probes. Observed signals are limited to live cells only; thus, information on cell viability also is provided. Moreover, as the reporter gene is passed on to the progeny cells, imaging of expanding cell populations becomes possible and correlates with an increase in the signal intensity. If the expression of the reporter gene is stable, labeled cells can be observed over their entire lifetime allowing for extended longitudinal studies upon serial infusions of reporter-specific probes (Figure 2). A successful, clinically applicable reporter gene and reporter probe combination depends on its mode of gene transfer, low immunogenicity, sensitivity of detection, pharmacokinetically optimized reporter probes, broad availability and feasibility, and low costs.

## Genetic Engineering Approaches

While engineered T cells are currently generated using randomly integrating viral vectors, this approach can result in variegated gene expression, clonal expansion, oncogenic transformation, and transcriptional silencing (7, 8). Developments in CRISPR/cas9 technology provide a way to specifically insert genes to a chosen genomic locus (9, 10). This can potentially result in greater gene expression and can ensure insertion in a transcriptionally active or controlled site (11). Higher density of expressed reporter molecules should improve the limit of detection of the modified cells *in vivo*. Still, viruses, such as adeno-associated viruses, are used to introduce the transgene to allow for homologous recombination into the cut sites generated by cas9. Non-viral, randomly integrating methods of gene transfer are also being employed; most notably the Sleeping Beauty transposon system.

## Reporter-Based Imaging of CAR T Cells

Imaging modalities that employ radionuclides for positron emission tomography (PET) followed by single-photon emission computed tomography (SPECT) offer the greatest promise for deep tissue imaging in humans with highest sensitivity for detecting low levels of reporter gene expression (picomolar sensitivity) and appear the most suited for clinical use. PET and SPECT, co-registered with CT or MRI, enable non-invasive, repetitive, and quantitative imaging of radiotracers for whole-body visualization of cell trafficking. High spatial anatomical resolution is achieved when co-registered with CT or MRI. Prerequisites for reporter probes are that its isotope and its decay products are non-toxic to T cells and that the probe is stably captured by the T cells. Several reporter gene systems employing PET or SPECT in preclinical experiments have been reported. Enzymes, transporters, and cell surface proteins have been used. There has been very limited use of these reporter systems in patients to date.

Herpes simplex virus type-1 thymidine kinase (HSV1-tk) is used for tracking of IL13Rα2- and PSMA-CAR T cells in clinical trials (NCT00730613, NCT01082926, and NCT01140373). A patient with recurrent glioblastoma, who after gross total tumor resection, received HSV1-TK-expressing IL13Rα2-CAR T cell infusions (cumulative T-cell dose of 1 × 10^9^) into the postsurgical cavity (12). Fused PET/MR images after the patient was intravenously injected with 9-(4-[^18^F]Fluoro-3-hydroxymethylbutyl) guanine ([^18^F]-FHBG) showed tracer accumulation at the surgical cavity site of infusion as well as at a non-resected tumor site. Subsequently, the study was expanded to include seven additional patients confirming the feasibility of this approach (13). These first studies lay the foundation for clinical CAR T imaging while also revealing challenges for broadly applicable tracking approaches. The lesion uptake values were relatively low (SUV up to 0.8) and non-specific radiotracer uptake was also noted in one patient. Furthermore, HSV-tk is a viral protein and immune reactions have been observed in patients, limiting its broadly applicable use (14).

Human deoxycytidine kinase double mutant (hdCKDM), another enzyme-based pyrimidine-specific reporter gene of human origin, has been tested in a preclinical study with PSMA-CAR T cells using 2′-[^18^F]fluoro-5-ethyl-1-β-D-arabinofuranosyluracil ([^18^F]-FEAU) (15). PET/CT scans performed after CAR T cell injection showed uptake in the lung at the tumor site compared to controls not receiving CAR T cells.

*Escherichia coli*dihydrofolate reductase enzyme (eDHFR) has been used as a reporter for imaging of GD2-CAR T cells with [^18^F]-labeled trimethoprim ([^18^F]-TMP) (16). PET/CT on days 7 and 13 post CAR T cell administration showed localization of the engineered cells initially in the spleen followed by accumulation at the GD2+ tumors. A detection limit of 11,000 cells per mm^3^ was determined. Nevertheless, eDHFR is also of bacterial origin, raising concerns of immunogenicity in clinical application. In addition, an association between adverse reactions to TMP treatment in combination with sulfamethoxazole (SMX) appears to correlate with HLA-type and is thus an aspect that needs to be considered for eDHFRs use (17).

Human Sodium Iodide Symporter (hNIS) is a human reporter gene with demonstrated utility for the temporo-spatial monitoring of PSMA-CAR T cells in mice with low and high tumor burden, illustrating an oscillating pattern (18). A minimum of 15,000 engineered T cells could be detected *in vivo*. hNIS is compatible with clinical radiotracers, notably sodium pertechnetate ([^99m^Tc]O${}_{4}^{-}$) and iodine-124 (^124^I) for SPECT and PET. A hurdle in using hNIS as a reporter is the physiologic expression in normal tissues, such as thyroid, salivary and lacrimal glands, stomach and lactating breast, as well as the limited imaging window due to rapid efflux of the radio isotope with exogenous expression of hNIS (19).

Human Somatostatin Receptor Subtype-2 (SSTR2) reporter is of human origin and can be targeted with clinically employed radiotracers. SSTR2 was used for tracking intercellular adhesion molecule-1 (ICAM-1) CAR T cells in mice bearing anaplastic thyroid cancer with ^68^Ga-DOTA-D-Phel-Tyr3-Octreotide ([^68^Ga]^68^Ga-DOTATOC; DOTA: 1,4,7,10-tetraazacyclododecane-N,N′,N″,N′-tetra-acetic acid) (20). Anti-tumor response correlated with expansion and contraction of the CAR T cells. A detection limit of T cells infiltrating a tumor located in the lung with a minimum density of 0.8% or ∼4 × 10^6^ cells/cm^3^ with 95% specificity and 87% sensitivity was reported. However, SSTR2 is physiologically expressed in cerebrum, kidneys, and gastrointestinal tract as well as on various immune cell types including T cells, B cells and macrophages, thus, limiting not only specificity, but as well potentially interfering with immune function (21).

Human Prostate Specific Membrane Antigen (hPSMA) reporter is as well of human origin and its radiotracers are currently used in the clinic. It is expressed by prostatic epithelium, and extraprostatic tissues, including small and large bowel, proximal renal tubules and brain as well as prostate cancer and tumor neovasculature (22). Truncated PSMA, devoid of its signaling function, was used for imaging of CD19 CAR T cells in a Nalm6 leukemia mouse model with a detection limit of near 2000 cells (23). Furthermore, a heterogenous CAR T response despite similar conditions, similar CAR T numbers derived from murine tumors as reported in human biopsy specimen from the TRANSCEND trial, and lack of correlation between CAR T cell numbers in peripheral blood or the bone marrow and those derived from the tumor were noted.

DOTA-antibody reporter gene 1 (DAbR1) paved the way for a new concept in cell tracking by introducing a membrane-bound cell surface anti-DOTA antibody fragment, which binds to a small radiohapten (radiohapten-capture imaging) (24). DOTA is a chelate and commonly used in MRI contrast agents and radiometal conjugates with a well-established safety profile. Recently, our group has shown that CD19 CAR T cells expressing DAbR1 can be tracked with PET and SPECT and potentially, by targeting with a radio-theranostic probe, serve as a “kill switch” for ablation of clustered, engineered T cells (25). DAbR1 is a molecule without a natural analog, thus, offers exquisite specificity. However, the scFv is of murine origin, raising concerns of immunogenicity.

## Consideration for the Selection of Radiolabeled Probes

Characteristics relevant for the use of radiolabeled probes for reporter gene imaging involve (i) sensitivity and specificity, (ii) pharmacokinetics, (iii) radiation dosimetry, (iv) availability of the radioisotope and probe precursor, (v) ease of radiosynthesis, (vi) half-life, which ideally should match the physiologic process being studied, and (vii) immunogenicity. Several reporter probes are already in clinical use, such as radiolabeled SSTR- analogs and PSMA-compounds, which obviates the need for safety and toxicity studies and establishing optimized radiolabeling protocols. Other aspects relate to the choice of the radioisotope, its availability, as well as its intrinsic characteristics regarding its radiation emissions that determine imaging performance and dosimetric profile. For example, the radiometal ^68^Ga (*t*_1/2_ = 68 min) is dosimetrically favorable, widely available from a ^68^Ge/^68^Ga generator and less expensive than other PET imaging isotopes such as ^64^Cu (*t*_1/2_ = 12.7 h), ^86^Y (*t*_1/2_ = 14.7 h), ^89^Zr (*t*_1/2_ = 3.3 days) which are cyclotron-produced. However, the short radioactive half-life of ^68^Ga limits its use to probes with rapid pharmacokinetic (e.g., small molecules and peptides). Longer half-life positron emitters such as ^89^Zr provide more optimal pharmacokinetics for monitoring cellular transit *in vivo*, into tumors and throughout blood and tissues over multiple days.

## Potential for Radiotheranostic Targeting

Given the promise of emerging reporter gene technologies and the power of radiotheranostic targeting, it is reasonable that the reporter gene approach will be adapted to combine both a platform for imaging and radiotherapy. Delivery of therapeutic isotopes via T cells can serve two purposes: (i) *in vivo* depletion of T cells in the case of toxicity (a “kill switch”) and (ii) to deliver a cytotoxic dose of radiation to residual tumor cells. T cell-based delivery of radiotherapy takes advantage of the unique capabilities of the T cells to home to, infiltrate and expand at tumor sites throughout the *entire* body. Combining quantitative imaging and engineering approaches (such as determination of sites of reporter molecules expressed per T cell) enabling dosimetric calculations prior to the administration of the therapeutic compound could pave the way for precision medicine also based on *radiotheranostic* T cell delivery.

## Conclusion and Outlook

The recent advance in adoptive T cell therapies and continued research in improving safety and outcomes for broader and larger patient population subsets requires parallel advances in non-invasive monitoring to facilitate preclinical development, clinical trials, prognosis and toxicity prevention. As compared to typical drugs or antibody therapeutics in which a dose is administered and its clearance predicted, these agents are living drugs; they can proliferate logarithmically and may persist for years within the patient, making traditional pharmacokinetic studies and predictions inadequate. The dose administered may not correlate directly with the final amount of the T cells at the tumor site or the site of tissue-toxicity. Real time monitoring of biodistribution is essential for understanding the mode of actions resulting in anti-tumor efficacy and tissue-toxicity. Without such imaging technology, the advance of these new cellular therapies will be slowed, the ability to predict outcomes and toxicities will be hampered, and the cost of clinical trials will be much larger.

One challenge is the detection of a small number of cells per unit volume inside a patient. Here, PET offers the greatest promise for deep tissue imaging in humans as the modality with the highest sensitivity, as compared to optical methods or MRI. Detection of few engineered cells is also dependent on the specificity of the reporter gene/probe combination for the targets of interest, in order to avoid non-specific tracer accumulation, resulting in decreased signal-to-noise ratio. In addition, safety, toxicity, immunogenicity and cost of the production of the reporter gene/probe combination need to be considered. In consideration of all the options, reporter gene-based cell tracking offers the distinct advantages of quantitative, non-invasive, long-term, real-time *in vivo* monitoring of cell trafficking in the entire body and has the future potential for radiotheranostic tumor and T cell ablation.

For example, a broadly applicable reporter gene would enable comparison between various constructs and offer insight into why some T cell therapies fail in the clinic, while others succeed. Early off-site targeting, such as accumulation of CD19 CAR T cells in the brain and cerebrospinal fluid, which may be a key factor involved in serious neurologic toxicity (26), or in the heart, causing lethal cardiomyositis (27) could enable mitigation of toxicity and increasing safety.

Important features of such a reporter gene/probe system would be (i) the ability to be applicable to a broad range of cellular therapies in a modular form, (ii) the ability to make use of a number of different radioactive probes, each matched to fit the clinical indication and time-course of the cells’ trafficking pattern, (iii) non-toxic and low or minimal immunogenicity of the reporter gene and probe, with (iv) low or no expression of reporter genes in normal tissues, (v) small reporter gene size, and (vi) non-enzymatic to enable direct quantitation of cell number. In addition, a non-immunogenic reporter gene/probe system would be optimal to allow repeated use and prevent toxicity. The probe and its metabolites should be safe so as to not confound the development of the therapy or its clinical use. Given the high cost of producing each of these cellular drugs for patients, the design of a reporter gene should be universal and applicable to multiple adoptive cell types, rather than selective for a single type of cancer, a single type of cellular agent or a single patient. While this perspective focuses on reporter gene use for engineered T cell therapies, a “universal” reporter gene designed for this purpose, could also be for any type of adoptive cellular therapy, including stem cell transplants.

Interdisciplinary efforts to realize the power of cell tracking during adoptive cell therapies in animals are essential to build the infrastructure and pave the way for pioneering clinical trials monitoring cellular kinetics in humans. HSV-tk is the only T-cell reporter gene reported in patients. Despite its caveats, these initial pilot studies represent a major breakthrough in the field of cellular tracking and prove the feasibility of using reporter gene imaging as a non-invasive method for tracking and monitoring CAR T cell localization in patients.

## Data Availability Statement

The original contributions presented in the study are included in the article/supplementary material, further inquiries can be directed to the corresponding author.

## Author Contributions

SK, MD, LC, and DS wrote the manuscript. DS initiated the project. DS and SL provided overall guidance. LC generated figures. All authors edited, reviewed, and contributed to the development of the final manuscript and approved the submitted version.

## Conflict of Interest

Memorial Sloan Kettering Cancer Center has filed for patent protection on behalf of DS, MD, SK, and SL for inventions related to work described in this manuscript. DS is an advisor to, or owns equity in, IOVA, PGNX, Eureka Therapeutics, CoImmune, PFE, that work in areas related to this manuscript. SL reports receiving commercial research grants from Genentech, Inc., WILEX AG, Telix Pharmaceuticals Limited, and Regeneron Pharmaceuticals, Inc.; holding ownership interest/equity in Y-mAbs Therapeutics Inc., and Elucida Oncology, Inc., and holding stock in ImaginAb, Inc. SL is the inventor and owner of issued patents both currently unlicensed and licensed by Memorial Sloan Kettering Cancer Center to Samus Therapeutics, Inc., Y-mAbs Therapeutics Inc., and Elucida Oncology, Inc.; is or has served as a consultant to Cynvec LLC, Eli Lilly and Co., Prescient Therapeutics Limited, Advanced Innovative Partners, LLC, Gerson Lehrman Group, Progenics Pharmaceuticals, Inc., and Janssen Pharmaceuticals, Inc. The remaining author declares that the research was conducted in the absence of any commercial or financial relationships that could be construed as a potential conflict of interest.
